# Positive predictive value of noninvasive prenatal testing for sex chromosome abnormalities

**DOI:** 10.1007/s11033-022-07754-x

**Published:** 2022-08-12

**Authors:** Nan Guo, Meiying Cai, Min Lin, Huili Xue, Hailong Huang, Liangpu Xu

**Affiliations:** Medical Genetic Diagnosis and Therapy Center, Fujian Maternity and Child Health Hospital, College of Clinical Medicine for Obstetrics & Gynecology and Pediatrics, Fujian Medical University, Fujian Key Laboratory for Prenatal Diagnosis and Birth Defect, Fuzhou, China

**Keywords:** Genetic counseling, Karyotype analysis, Pregnancy, Single nucleotide polymorphism array

## Abstract

**Background:**

Early and intermediate serological screening cannot detect sex chromosome abnormalities. Currently, noninvasive prenatal testing (NIPT) is the only procedure available for screening such disorders; however, its use is controversial.

**Methods and Results:**

A total of 47,855 pregnant women underwent NIPT at our referral center from January 2014 to December 2020. Of the 314 patients with a positive NIPT indicating sex chromosome abnormalities, 260 were screened via karyotype analysis and single nucleotide polymorphism (SNP) array after amniotic fluid extraction; 96 cases were confirmed. Karyotype analysis and SNP array were consistent in the diagnosis of 88 out of the 96 fetuses. The positive predictive value (PPV) for sex chromosome abnormalities was found to be 36.9%. The PPV in patients aged 30–34 years was significantly higher than that in patients aged < 30 years. No statistically significant difference was observed on the PPV among patients with or without previous adverse pregnancy outcomes. Moreover, 83 women carrying fetuses were diagnosed with a sex chromosome abnormality terminated their pregnancy.

**Conclusions:**

Improvements in detection and analytical technologies are needed to increase the accuracy of sex chromosome abnormalities detection. Pregnant women with a positive NIPT for these abnormalities may require invasive diagnostic procedures such as karyotype analysis and SNP array for better genetic counseling.

## Introduction

Sex chromosome abnormalities refer to abnormalities in the structure or number of sex chromosomes (Y or X). Common clinical sex chromosome abnormalities include Turner syndrome, Klinefelter syndrome, 47, XXX syndrome, mosaicism, and structural abnormalities. The incidence of sex chromosome abnormalities is approximately 1/300–1/400 [1]. Clinical manifestations are diverse, with some patients having no abnormal phenotype, and others showing height abnormalities, organ structural abnormalities, intellectual disability, neurological dysfunction, and infertility caused by dysplasia of secondary sexual characteristics [2, 3].

Lo et al. [4] first discovered cell-free fetal DNA in the plasma of pregnant women in 1997. This discovery led to the development and implementation of noninvasive prenatal testing (NIPT) for screening the most common trisomies and sex chromosomal anomalies. It is a method to obtain information on fetal chromosomes by deep sequencing free DNA from the maternal peripheral blood using large-scale parallel sequencing technology. NIPT has been successfully used to detect trisomy 21, trisomy 18, and trisomy 13 with a sensitivity of 100%, 100%, and 91.9%, respectively, and a specificity of more than 99% [5–9]. Therefore, these three syndromes are the main target diseases when performing NIPT.

Sex chromosome abnormalities are not always associated with clinical features or sonographic abnormalities in the prenatal period. Additionally, early and intermediate serological screening cannot detect sex chromosome abnormalities. Currently, NIPT is the only available procedure for screening fetal sex chromosome disorders and is, therefore, a very valuable tool in clinical practice. However, its use for the prenatal screening of these abnormalities is still controversial [10–13]. In the present study, 47,855 pregnant women who underwent NIPT at our referral center from January 2014 to December 2020 were retrospectively studied. Amniotic fluid was obtained from women whose NIPT indicated a high risk for a sex chromosome abnormality; these samples were subjected to karyotype analysis and a single nucleotide polymorphism (SNP) array. A positive predictive value (PPV) was calculated to investigate the clinical application value of NIPT in screening for fetal sex chromosome abnormalities.

## Materials and methods

### Patients

A total of 47,855 pregnant women who underwent NIPT in the Prenatal Diagnosis Center of Fujian Maternal and Child Health Hospital from January 2014 to December 2020 were retrospectively studied. The age range of the women was 18–48 years and their gestational age was in the range of 13 + 1–26 + 6 weeks. The inclusion criterion for this study was single pregnancy. The exclusion criteria were a family history of a chromosome abnormality or a previously diagnosed fetal abnormality, women who had received a blood transfusion or undergone a transplantation operation or stem cell therapy within one year, and those who had malignant tumors.

## Noninvasive prenatal testing

Informed consent was obtained from the pregnant women and their families before the study was started. Peripheral blood (10 mL) was collected from each patient and placed in tubes containing ethylenediaminetetraacetic acid. Plasma was separated from the blood within 4 h. A QIAamp Circulating Nucleic Acid Kit (Qiagen, Hilden, Germany) was used to extract free DNA from the plasma samples. A DNA library was constructed by terminal repair, splicing, and PCR, and quantified using Qubit. Fetal chromosome aneuploidy kits (T21, T18, T13) were used to detect DNA migration from peripheral blood of pregnant women usingthe Next Seq CN500 (Berry Genomics Corporation, Beijing, China) sequencing platform. The sequence alignment software BWA map was used to align sample sequences to the reference sequence map of the human genome. The proportion of each chromosome (% ChrN) and the Z value for each chromosome (cutoff: | Z |= 3) were calculated. | Z |> 3 indicates a high risk of aneuploidy abnormalities and requires further evaluationby invasive prenatal tests. | Z |≤ 3 indicates low risk (i.e., low likelihood of fetal chromosomal aneuploidy) and requires clinical follow-up.

## Karyotype analysis

The standard methods of karyotype analysis were used. Karyotypes were named according to the 2016 edition of the International System for Human Cytogenomic Nomenclature. A total of forty karyotypes were counted for each sample, of which five were analyzed; twenty additional karyotype counts and analyses were performed in the case of detected abnormalities.

## SNP array

DNA samples were digested, ligated, amplified, purified, labeled, hybridized, washed, and scanned according to the experimental standard operating procedures provided by Affymetrix, Inc. (Santa Clara, CA, USA). Data were analyzed using the matching Chromosome Analysis Suite software. SNP array results were further analyzed using the relevant database to determine the nature of copy number variation (CNV), which can be divided into three categories [14, 15] as follows: pathogenic, benign, and unknown clinical significance (VUS). VUS CNVs can be further divided into potentially pathogenic, potentially benign, and clinically unknown. For VUS CNVs, SNP array on peripheral blood samples of fetal parents was performed to further clarify the nature of CNV in combination with pedigree analysis.

## Pregnancy outcome follow-up

All cases were followed up by telephone to evaluate fetal development, pregnancy outcome, and postpartum growth and development.

### Statistical analysis

SPSS 25.0 software (IBM Corp., Armonk, NY, USA) was used for the statistical processing of data. Enumeration data were expressed as rate (%). The chi-square test was used to compare data between groups. Statistical significance was considered at P < 0.05 in a bilateral test.

## Results

### Noninvasive prenatal testing

We found that out of the 47,855 pregnant women, 314 were at a high risk of fetal sex chromosome abnormalities, with a positive screening rate of 0.66%.

## Karyotype and SNP analysesfor fetuses with positive NIPT

Out of the 314 women who had a NIPT indicating a high risk for a sex chromosome abnormality, a total of 54 cases could not be traced. Therefore, amniotic fluid samples were extracted from 260 patients for karyotype and SNP array analyses. Of these patients, 96 were diagnosed with sex chromosome abnormalitiesat a total PPV of 36.9%. Of the diagnosed abnormalities, 47, XYY had the highest PPV (52.9%), whereas 45, X had the lowest (21%) (Table1). The ninety-six cases of confirmed sex chromosome abnormalities included thirty-three cases of Klinefelter syndrome, eighteen cases of 47, XYY syndrome, seventeen cases of 47, XXX syndrome, two cases of 45, X Turner syndrome, fifteen cases of mosaic Turner syndrome, two cases of 47, XXY mosaic, seven cases of structural abnormalities in sex chromosomes, one case of 45, X[34]/47, XXX[11], and one case of 45, X[41]/47, XXY[30]/46, XY[2].

**Table 1:** PPV of various types of sex chromosome abnormalities

NIPT-based diagnosis	Number of positive NIPTs	Number of true positives	PPV (%)
45,X	100	21	21
47,XXX	45	19	42.2
47,XXY	81	38	46.9
47,XYY	34	18	52.9
Total	260	96	36.9

*PPV* positive predictive value, *NIPT* noninvasive prenatal testing

Furthermore, the results of karyotype analysis and SNP array we consistent for 88 out of the 96 cases, at a complete detection rate of 91.7%. Six cases were inconsistent, including two of chromosomal microdeletions and four of low-level mosaicism of sex chromosomes. Unfortunately, the SNP array cannot detect low-level mosaicism of sex chromosomes, and karyotype analysis cannot detect chromosomal microdeletions (Table 2).

**Table 2:** Inconsistent results betweenkaryotype analysis and SNP array

Case	Karyotype analysis	SNP array	NIPT
1	45,X[7]/46,XX[99]	Normal	45,X
2	45,X[7]/46,XX[73]	Normal	45,X
3	45,X[7] /46,XX[72]	Normal	45,X
4	45,X[41]/47,XXY[30]/46,XY[2]	Normal	45,X
5	Normal	arr[hg19] Yq11.223q11.23(25,863,808–27,609,692) × 0	47,XXX
6	Normal	arr[hg19] Xq24q25(118,395,148–125,416,121) × 1	45,X

*PPV* positive predictive value, *NIPT* noninvasive prenatal testing

## Comparison of PPVs in different age groups

The pregnant women who had a positive NIPT, indicating a high risk for a sex chromosome abnormality, were divided into three groups according to their ages as follows: < 30, 30–34, and ≥ 35 years groups. The PPV for sex chromosome abnormalities was different between the three age groups (Table 3); it was highest for the 30–34-year group followed by the ≥ 35-year group. Statistical analysis indicated that the PPV for sex chromosome abnormalities in the 30–34-year group was significantly higher than that in the < 30-year group (X^2^ = 5.252, P < 0.05).

**Table 3:** Comparison of PPVs of sex chromosome abnormalities amongdifferent age groups

Age group	NIPT-positive	True positive	PPV (%)
< 30	91	64	29.7
30–34	91	49	46.2
≥ 35	78	51	34.6
Total	260	164	36.9

*PPV* positive predictive value, *NIPT* noninvasive prenatal testing

## Comparison of PPVs based on the frequency of prior adverse pregnancy outcomes

The pregnant women were divided into another set of three groups according to the frequency of past adverse pregnancy outcomes as follows: no adverse pregnancy outcome, one adverse pregnancy outcome, and two or more adverse pregnancy outcomes. “No adverse pregnancy outcomes” indicates no prior pregnancy losses; “adverse pregnancy outcomes” indicate the occurrence of previous miscarriages. No statistical difference was observed for the PPVs for sex chromosome abnormalities among the three groups (X2 = 1.358, P > 0.05; Table 4).

**Table 4:** Comparison of PPVs of sex chromosome abnormalities based on the frequency of adverse pregnancy outcomes

Frequency of adverse pregnancy outcomes	NIPT-positive	True positive	PPV (%)
0	146	95	34.9
1	59	38	35.6
≥ 2	55	31	43.6
Total	260	164	36.9

*PPV* positive predictive value, *NIPT* noninvasive prenatal testing

## Pregnancy outcomes for women with positive NIPT for sex chromosome abnormalities

A total of 256 women out of the 260 patients who had a positive NIPT for sex chromosome abnormalities were followed up successfully; however, three women were lost to follow-up, which gave a follow-up rate of 98.5%. Furthermore, 83 pregnancies out of the 96 cases of confirmed sex chromosome abnormalities were terminated, giving an overall termination rate of 86.5%. However, four pregnant women with normal karyotype and SNP array results had a miscarriage. A total of 169 normal deliveries were recorded, among which 8 fetuses had 47,XYY syndrome, 3 had low-level mosaicism, 2 had balanced translocations, and 156 had normal karyotype and SNP array results.

## Discussion

A total of 314 out of the 47,855 pregnant women who underwent NIPT was found to be at a high risk of fetal sex chromosome abnormalities, with a positivity rate of 0.66%. This is consistent with the findings of Reiss et al. [16], who obtained a positivity rate of 0.63% for 2851 women with single pregnancies. The accuracy of NIPT in predicting fetal sex chromosome abnormalities has always been controversial, with a reported PPV range of 38–50% in the literature [16–19]. Bin Yu et al. [20] screened fetal sex chromosome abnormalities and reported a PPV of 54.54% through NIPT. In another study by Zhang et al. [19], 275 pregnant women underwent NIPT and the positivity rate from screening for sex chromosome abnormalities was 0.55% with a PPV of 54.54%. In the present study, the obtained PPV was 36.9%, which was significantly lower than the values reported in the reports above; 47, XYY had the highest PPV (52.9%), whereas 45, X had the lowest (21%). Studies assessing the PPV of NIPT in the screening of sex chromosome abnormalities had different sample sizes and sequencing depths. Moreover, NIPT has a relatively low specificity in detecting sex chromosomes. This may be due to the X chromosome guanine-cytosine content deviation. Maternal age-related loss of the X chromosome could be another potential factor in false positive NIPT results for sex chromosome abnormalities. Moreover, the high homology of X and Y chromosomes is not conducive to discrimination compared withother chromosomes. Additionally, the Y chromosome has more similar segments, which reduces the sequencing noise ratio, and factors such as fetal chromosomal chimerism can lead to inaccurate analysis and high false positivity rates [21]. Therefore, it is necessary to further improve this technology to enhance the detection accuracy of abnormalities.

Karyotype analysis is the “gold standard” for cytogenetic diagnosis. It can detect all chromosomal aneuploidies and structural abnormalities that are visible under amicroscope. In the present study, of the 260 pregnant women who had a positive NIPT for sex chromosome abnormalities, 94 had their diagnosis confirmed by karyotype analysis, including 70 cases of aneuploidy, 19 cases of mosaicism, and 5 cases of structural abnormalities. SNP array confirmed 90 cases of sex chromosome abnormalities, including 70 cases of aneuploidies, 15 cases of mosaicism, and 5 cases of structural abnormalities. SNP array is a molecular genetics technology that was developed in recent years [22]. It has significant advantages such as the ability to detect chromosome microdeletions or microduplications that cannot be detected by karyotype analysis at the genomic level [23–25]. However, it cannot detect structural abnormalities of chromosomal balance and low-level mosaicism. In our study, the SNP array missed the detection of four cases of low-level mosaicism for sex chromosome abnormalities. There are many types of sex chromosome abnormalities; therefore, the SNP array cannot completely replace karyotype analysis. To determine the PPV of a sex chromosome abnormality, we suggest that both karyotype analysis and SNP array should be performed to obtain an accurate scientific and molecular genetic diagnosis for reproductive planning for the next pregnancy.

Studies have shown that the age of women is closely related to the occurrence of germ cell aneuploidy and that the incidence of germ cell aneuploidy increases as women increase in age [26, 27]. In the present study, the PPV for sex chromosome abnormalities in the 30–34-year group was significantly higher than that in the < 30-year group. This suggests that NIPT is more accurate for the detection of fetal sex chromosome abnormalities in women over the age of 30. However, the PPV for sex chromosome abnormalities in the ≥ 35-year group was higher than that in the < 30-year group but lower than that in the 30–34-year group. This might be because most pregnant women aged ≥ 35 years, being comparatively older, usually have their amniotic fluid drawn directly for prenatal analyses.

Most parents do not accept carrying fetuses with sex chromosome abnormalities to term after detailed genetic counseling. The termination rate after prenatal diagnosis of sex chromosome abnormalities varies greatly from 15.5 to 97.5% in different countries [28, 29]. Several factors influence the decision to terminate pregnancies with sex chromosome abnormalities; these are mainly related to sex chromosome category, the existence of ultrasound structural abnormalities, gestational age at prenatal diagnosis, age of parents, or desire for children, among other factors [30]. The type of sex chromosome abnormality is an important factor that affects pregnancy outcomes. Compared to 47,XXX, 47,XYY, and sex chromosome mosaics, 45,X and 47,XXY have more severe clinical phenotypes, including sexual dysplasia, infertility, and behavioral abnormalities. Therefore, women whose fetusesare diagnosed with 45,X or 47,XXY are more likely to terminate their pregnancies. Sex chromosome abnormalities with low-level mosaicism have less obvious clinical manifestations and better prognoses. In this study, eight fetuses with 47,XYY, three fetuses with low-level mosaicism for sex chromosomes, and two fetuses with balanced translocation were followed up after birth, and all showed normal phenotypes. However, this study had an obvious limitation. For the sensitive issue of fetal sex, increased or decreased sex chromosomes were not reported in our NIPT results. Unfortunately, we can't calculate PPVs for 45,X, 47,XXX, 47,XXY, 47XYY detection with distinct age ranges.

In conclusion, the accuracy of NIPT is affected by various factors during the screening of sex chromosome abnormalities, and the PPV is relatively low; further methodological improvements are required to enable an enhanced detection accuracy. Moreover, because there are many types of sex chromosome abnormalities, pregnant women with sex chromosome abnormalities who are screened by NIPT require further invasive procedures such as karyotype analysis and SNP array to verify diagnoses. Additionally, genetic consultants should have access to update information to provide guidance for patients and reasonable treatment plans when fetuses have sex chromosome abnormalities.

## Data Availability

The datasets used and/or analyzed during the current study are available from the corresponding author upon request.
